# Improvement of Circadian Rhythm of Heart Rate Variability by Eurythmy Therapy Training

**DOI:** 10.1155/2013/564340

**Published:** 2013-03-05

**Authors:** Georg Seifert, Jenny-Lena Kanitz, Kim Pretzer, Günter Henze, Katharina Witt, Sina Reulecke, Andreas Voss

**Affiliations:** ^1^Task Force Integrative Medicine in Pediatric Oncology, Department of Pediatric Oncology and Hematology, Otto-Heubner-Center for Pediatric and Adolescent Medicine (OHC), Charité-Universitätsmedizin Berlin, Augustenburger Platz 1, 13353 Berlin, Germany; ^2^Department of Medical Engineering, University of Applied Sciences Jena, 07745 Jena, Germany

## Abstract

*Background*. Impairment of circadian rhythm is associated with various clinical problems. It not only has a negative impact on quality of life but can also be associated with a significantly poorer prognosis. Eurythmy therapy (EYT) is an anthroposophic movement therapy aimed at reducing fatigue symptoms and stress levels. *Objective*. This analysis of healthy subjects was conducted to examine whether the improvement in fatigue symptoms was accompanied by improvements in the circadian rhythm of heart rate variability (HRV). *Design*. Twenty-three women performed 10 hours of EYT over six weeks. Electrocardiograms (ECGs) were recorded before and after the EYT trial. HRV was quantified by parameters of the frequency and time domains and the nonlinear parameters of symbolic dynamics. *Results*. The day-night contrast with predominance of vagal activity at night becomes more pronounced after the EYT training, and with decreased Ultralow and very low frequencies, the HRV shows evidence of calmer sleep. During the night, the complexity of the HRV is significantly increased indicated by nonlinear parameters. *Conclusion*. The analysis of the circadian patterns of cardiophysiological parameters before and after EYT shows significant improvements in HRV in terms of greater day-night contrast caused by an increase of vagal activity and calmer and more complex HRV patterns during sleep.

## 1. Introduction

Eurythmy therapy (EYT) is a mind-body therapy widely used in *anthroposophic medicine* in Europe. In a crossover study in healthy subjects, deeper relaxation and improved vagal activity of heart rate variability were found after a single session of EYT compared with training on an exercise bicycle [1]. This study shows on the basis of physiological measurements that EYT is a highly effective body therapy intervention with regard to achievement of states of deep relaxation and stimulation of heart rate variability (HRV). The study showed that despite identical levels of exertion, the vagal stimulation following EYT was qualitatively very different from that after exercise bicycle training. EYT is indicated for somatic diseases as well as for psychosomatic diseases such as states of stress, burnout, chronic fatigue, or insomnia. In addition to clinical experience and a number of anecdotal reports, there is some evidence in the literature that EYT can have positive effects in various chronic diseases [2, 3]. Heart rate variability has considerable potential to assess the role of autonomic nervous system (ANS) fluctuations in normal healthy individuals and in patients with various cardiovascular and noncardiovascular disorders [4]. The conventional statistical methods calculating variance and power spectral analysis of HRV are most often used [5]. The high-frequency (from 0.15 to 0.4 Hz) fluctuations of heart rate are determined by respiration representing autonomic neural fluctuations and central blood volume alterations [6]. These high-frequency fluctuations are modified by the phenomenon called Respiratory gate [7]. The low-frequency (from 0.04 to 0.15 Hz) fluctuations of heart rate have been proposed to be derived from the arterial pressure Mayer waves, whose major determinant is considered to be sympathetic vasomotor activity [6]. The very-low-frequency fluctuations (below 0.04 Hz) have been attributed to the renin-angiotensin system, other humoral factors, and thermoregulation. For example, it has been shown that the reduction in HRV correlates reliably with the subjective quality of sleep in patients suffering from CFS [2, 3]. 

Methods of HRV analysis based on nonlinear system theory and beat-to-beat dynamics have gained recent interest as they may reveal dedicated changes of heart rate time series [5]. Several types of different fractal scaling measures, power-law analyses, complexity measures, measures of symbolic dynamics, turbulence, and deceleration capacity of heart rate have been studied in various patient populations. These methods of analysing HRV aim to assess qualitative properties rather than the magnitude of the signal [5].

Many stressors such as stress at work, anxiety, and depression are associated with reduced HRV and/or increased sympathetic activity and decreased vagal activity. Loss of the protective vagal activity is associated with an unfavourable prognosis with regard to various cardiovascular diseases and sudden cardiac death [8]. Various changes in HRV or the autonomic nervous system are seen particularly in mental and psychosomatic diseases. It should be emphasised that the reduction in HRV correlates reliably with the subjective quality of sleep in patients suffering from CFS [2, 3]. Therefore physiological measurements of HRV are particularly suitable for investigation of complex body therapies such as yoga [9], meditation [10], or EYT [1] which are reported to have a regulating effect on stress and night-time sleep [11, 12].

This study is based on previously published data from a study involving 68 healthy, moderately stressed adults (mean age: 42.2; SD: 8.2) which showed that a 6-week course of EYT training can significantly improve quality of life and stress coping in comparison with an untreated control group. The data also showed a significant decrease in the initially pronounced fatigue symptoms [2, 3].

In this continuative analysis of the study, we tested the hypothesis that the clinical improvements in the fatigue symptoms would also be reflected in a changed chronobiological circadian pattern of HRV. Therapeutic experience shows that EYT has a positive impact on night-time sleep, which might thus explain the published improvements in the fatigue symptoms [11]. The rationale for the study hypothesis was the demonstrated connection between reduced quality of night-time sleep and reduced HRV in patients with CFS symptoms [2, 3]. Circadian chronobiological rhythm in this context means particularly the fluctuation between the different physiological states of the ANS during the day and during the night. In order to explore this question, we undertook this detailed analysis of a cohort completing a six-week period of EYT training. 

## 2. Methods

### 2.1. Subjects

The study group comprised 23 healthy women (mean age: 45.97), who were recruited from a population of teachers and nursery teachers. Each subject had an intervention of two treatment sessions per week over a period of 6 weeks. Before beginning the YET, the subjects completed a baseline visit involving performance of a psychometric test. In addition, the level of activity was controlled by requesting from the subjects to refrain from sport or other unusual physical activity and from consumption of alcohol or stimulants. The 23 women consented to recording of a 24-hour electrocardiogram (ECG) before and after the EYT. The study was approved by the Ethics Committee of the Charité-Universitätsmedizin Berlin.

### 2.2. EYT Intervention

Two EYT therapists conducted the training. The exercises were learned gradually over 2 weeks and repeated in each treatment unit (45 minutes) so that the subjects were able to perform them independently later. The sequence followed an identical pattern throughout the 6 weeks. The rhythm exercises are special EYT exercise sequences performed on the basis of the speech rhythms of verses or poems. Lyrical patterns or rhythms such as the hexameter (the classical metre of epic poetry), dactyl (a metre consisting of one heavy (long) syllable followed by two light (short) syllables), anapest (reverse of dactyl), or other variations are used. These involve rising and falling speech rhythms translated into movement and acceleration and deceleration. In addition, ball exercises, rod exercises, stepping exercises, and central speech sound exercises with consonants (L and M) formed a part of each treatment unit. The essential element of these movement exercises is the alternation between tension and relaxation and a harmonious relationship between inhalation and exhalation. For the emotional (soul) sector, exercises on spatial orientation and boundary formation and geometric exercises for inner structuring (iamb and trochee) were used. This was followed by speech sound exercises with vowels O, E, and U and soul exercise with E. Concluding exercises with a meditative character in combination with physical movement used phrases such as “I have calm within myself, I have within me the forces that give me strength.” These EYT exercises were chosen because we believed that this combination would have some impact on HRV as well as on the clinical outcome parameters concerning quality of life and stress.

### 2.3. HRV

From the raw 24 h Holter ECG data, 4 h of day time (10.00 a.m. to 02.00 p.m.) and 4 h of night time (00.00 to 04.00 a.m.) were calculated, and time series of heart rate (tachogram) consisting of beat-to-beat intervals (BBIs) were extracted. The participants were instructed to sleep normally during the night and to refrain from alcohol and stimulants. This was checked by means of activity protocols. To obtain normal-to-normal (NN) heart beat time series, ectopic beats and disturbances or artifacts were interpolated in the given BBI series applying an adaptive variance estimation algorithm [13].

On the basis of the guidelines of the Task Force of the European Society of Cardiology [4], the following HRV standard indices from the time and frequency domains were calculated from the tachograms:

#### 2.3.1. Time Domain

Consider the following:

- meanNN = mean value of NN interval time series (ms);
- sdNN = standard deviation of NN interval time series (ms);
- rmssd = square root of the mean squared differences of successive N intervals (ms).

#### 2.3.2. Frequency Domain

Power spectra were calculated by applying fast Fourier transform with a Blackman Harris window of equidistant NN interval time series. To obtain equidistant time series, the tachograms were linearly interpolated and resampled at 10 Hz. From the spectrum, the following “standard” [9] indices were calculated:

- ULF = power in the “ultralow” frequency band < 0.003 Hz;
- VLF = power in the “very low” frequency band 0.003–0.04 Hz;
- LF = power in the “low” frequency band 0.04–0.15 Hz;
- HF = power in the “high” frequency band 0.15–0.4 Hz;
- LF/HF = ratio of LF to HF power;
- LFn = LF power normalized as LFn = LF/(LF + HF);
- HFn = HF power normalized as HFn = HF/(LF + HF);
- LF/*P* = LF power normalized by the total power *P; *
- HF/*P* = HF power normalized by the total power *P. *

In addition, we calculated the following indices:

- *P* = total power in the band 0.00–0.4 Hz;
- (ULF + VLF)/*P* = power in the frequency band 0.0–0.04 Hz (ULF + VLF) normalized to the total power (for modified version, see [14]).

#### 2.3.3. Nonlinear Dynamics

*(a) Symbolic Dynamics (SD).* Symbolic dynamics is a nonlinear method which describes the global short- and long-term dynamics of beat-to-beat variability on the basis of symbolization. Methods of nonlinear symbolic dynamics analysis [4, 13] have been shown to be sufficient for the investigation of complex systems and describe dynamic aspects within time series. The method applied in this study (there are different approaches for deriving symbolic dynamics from a time series) is described in detail elsewhere [13], and in the following, only a brief introduction is given. The NN beat-to-beat interval time series are converted into an alphabet of four predefined symbols (0, 1, 2, and 3) [14] according to the transformation rules based on consecutive comparison of successive beat-to-beat intervals. The symbols “0” and “2” reflect slight deviations (increase or decrease) from the mean NN interval, and the symbols “1” and “3” reflect stronger deviations (increase or decrease over the mean NN interval and in addition over a predefined limit). Then, the symbol strings are transformed into word series where each word consists of three successive symbols. This leads to a range of 64 different word types (000,001,…,333). Then, we estimate from the word distribution the probability of occurrence (pWxxx) of each word type (xxx = 000, 001,…,333) within NN interval time series. As an example, the word type 321 [6] (consisting of the successive symbols 3, 2, and 1) represents sequences of two decreasing beat-to-beat intervals followed by an increasing one and can be interpreted as a fast heart rate downregulation after a heart rate increase.

The indices pTH1 to pTH20 [15] represent the number of words with a probability of occurrence higher than a specific threshold (e.g., pTH7: 7%). 

*(b) Short-Term Symbolic Dynamics (STSD).* The short-term symbolic dynamics is a modification procedure of symbolic dynamics and is introduce by Porta et al. [16]. This method used in windowed 300 beat-to-beat intervals. The time series is transformed into six symbol sequences (0–5). Each sequence of three symbols formed words which are summarized in word families. As follows, an example is explained The sequence pattern 1 2 1 or 2 4 3 implies a maximum in the middle and represents peak.

*(c) Multiscale Entropy*. The multiscale entropy (MSE) analysis is a method to characterize the complexity of a finite length of interbeat (RR) interval time series. This method is based on sample entropy (SampEn), which quantifies the irregularity of a time series and estimates the conditional probability of similarity between two sequences of *m* and *m* + 1 consecutive datapoints. The discrete time series {*x*_1_,…, *x*_*i*_,…, *x*_*N*_} is transformed into different time scales, whose values are *τ* = 1, 2,…, 20  (scale  1,…, scale  20). For scale 1, the time series is simply the original time series. Scale 2 is generated by calculating the mean value of 2 successive values of the original times series with nonoverlapping windows. For each additional scale *τ*, a successive average of *τ* values of the original time series is determined. Finally, the sample entropy for each coarse-grained time series is calculated. Due to the averaging of a successively increasing number of values, the sample entropy of small scales (1–5 beats) is a measure for short-term complexity, whereas longer scales (6–20 beats) represent long-term complexity [17]. Thus, it estimates correlations at multiple (time) scales. The algorithm is described in detail elsewhere [16]. The MSE method demonstrated that healthy HRV is more complex than pathological HRV [16]. 

### 2.4. Statistics

The analysis was performed at first with the Friedman test as a nonparametric method. The nonparametric Wilcoxon rank-sum test was applied for the statistical analysis of two dependent samples. The evaluation was performed on the basis of the HRV indices of each subject before and after EYT separately for day and night times. Values of *P* < 0.05 (*) were regarded as statistically significant, values of *P* < 0.01 (**) as highly significant, and values of *P* < 0.001 (***) as most significant. Considering the day-night contrast, the significance test was applied between day time and night time before and after EYT. 

A two-factor ANOVA with repeated measures normalising the data (by transformation) before analysis was applied to investigate (1) the general influence of EYT (factor time before/after EYT), (2) the influences of EYT on day and night times before and after EYT (factor phase), and (3) the interaction between the factors time and phase. 

## 3. Results

### 3.1. Heart Rate Variability

The ECG data from 23 healthy women were evaluated before and after EYT. Several indices showed changes in the autonomic regulation between day time and night time. Only indices that showed significant changes (*P* < 0.05) between day and night times before EYT but not after EYT and vice versa and indices that are standard HRV indices (time and frequency domains) in various other studies were considered.

**Figure 1:**
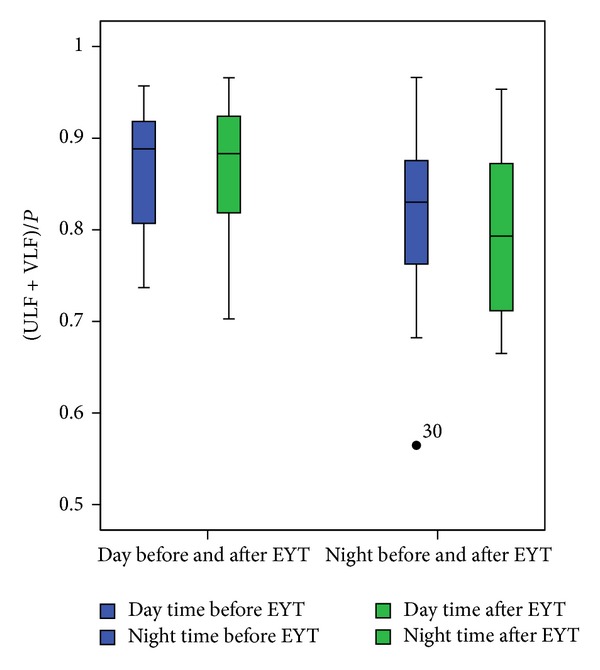
Box plots of the HRV index (ULF + VLF)/*P* for day time before and after EYT and night time before and after EYT.

**Figure 2:**
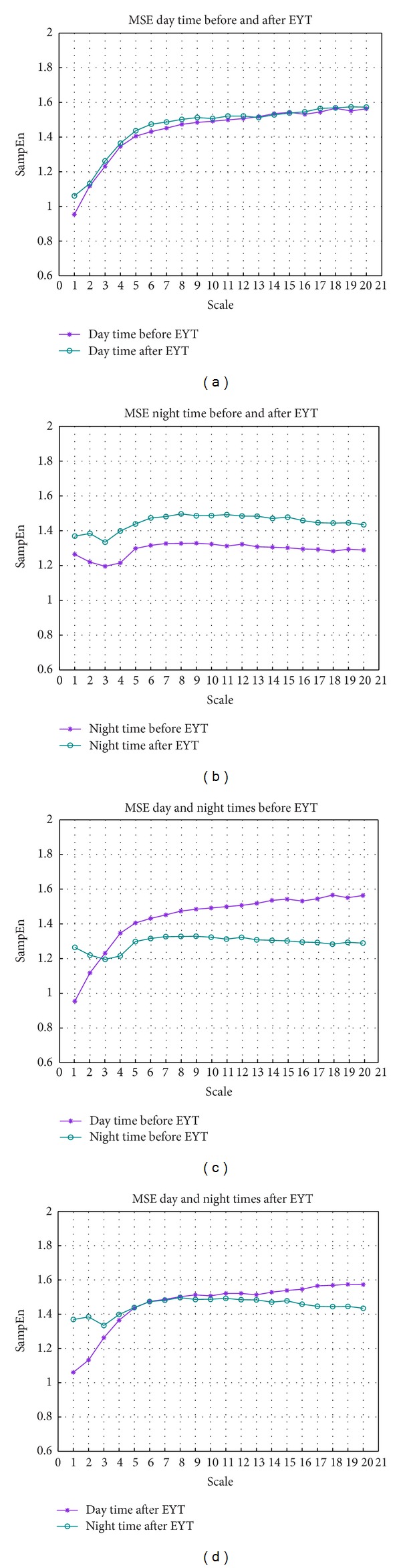
Behaviour of MSE indices before and after EYT. The sample entropy (SampEn) (*y*-axis) is plotted over the scales (*x*-axis). (a) MSE during day before and after EYT. (b) MSE during night before and after YET. (c) MSE during day and night before EYT. (d) MSE during day and night after EYT.

The mean beat-to-beat interval (meanNN) was significantly different between day and night (increased during the night); however, this difference remained practically unchanged before and after EYT (Table 1). Table 1 shows the mean values and standard deviations of the indices. The standard deviation (sdNN) shows no significant differences between day and night even after EYT. LFn decreases during the night after YET, whereas HFn increases. LF/HF is not significantly changed by EYT but exhibits a shift towards lower values (trend) confirming the LFn and HFn results. ULF and the normalized (ULF + VLF)/*P* decreased significantly during the night after EYT (Figure 1). The indices from symbolic dynamics word type pW232 and pTH7 are generally increased during the night. While pW232 shows significant differences between day and night after EYT, the index pTH7 changed in the opposite direction (significant between day and night before EYT and nonsignificant after EYT). Further, the index mean_huegel from short-term symbolic dynamics is generally increased in the night. The day-night difference after EYT is increased, so that the significance level changed from nonsignificant to *P* < 0.01. Finally, the indices scale 2 and scale 20 from MSE are generally increased during the night time after EYT (Figure 2(b)), whereas they are not changed during day time (Figure 2(a)). The differences between day and night, however, are significantly increased after EYT in scale 2 and decreased in scale 20 (Figures 2(c) and 2(d)) after EYT. 

**Figure 3:**
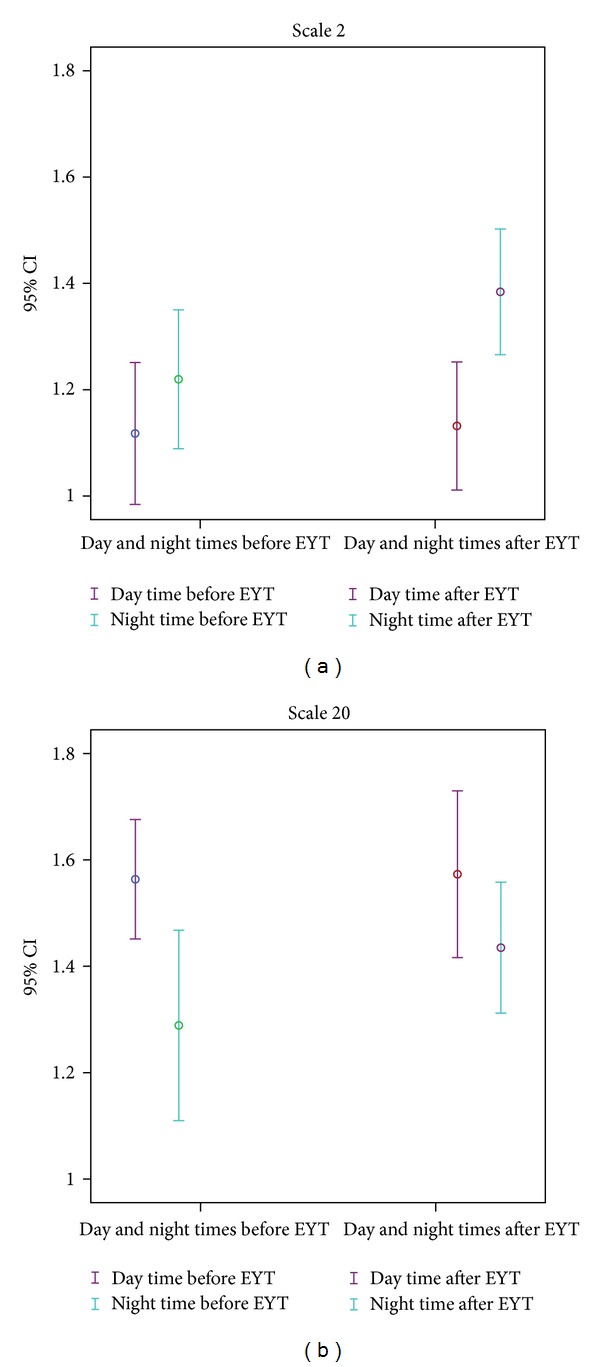
Error bar of the indices scale 2 and scale 20 comparing day and night times before and after EYT (confidence interval 95%).

Figure 3 shows the error bar of the indices scale 2 and scale 20 of the day and night times before and after EYT (confidence interval 95%). 

The ANOVA analysis revealed highly significant phase dependencies in all indices with the exception of SDNN and LF/HF. No significant time dependencies and interactions could be found; however, a clear trend (*P* < 0.15) especially in the indices scale 2 and scale 20, pW232, and mean_huegel could be observed.

**Figure 4:**
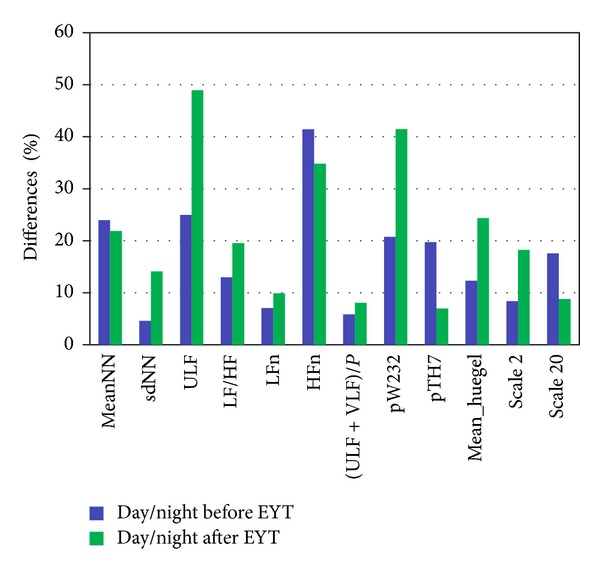
Differences of selected HRV indices between day and night times before (blue) and after (green) EYT as percentage changes.

The differences between day and night times before and after EYT as percentage changes are demonstrated in Figure 4.

## 4. Discussion

The general aim of this study was to examine the physiological effects of an innovative intervention on day-time and night-time HRV and thus to provide a basis for optimization and explanation of new treatment approaches such as EYT. In this study of a cohort of 23 women completing a 6-week course of YET training, we analysed the 24-hour ECGs with respect to the circadian pattern of the HRV changes. The most important finding is that there are significant changes between the day and night times before EYT and the day and night times after EYT (Figure 4). 

The 23 women in the study showed an increase in vagal activity (increased difference of HFn after EYT) and in the complexity of autonomous regulation (pW232, pTH7, mean_huegel, and scales 2 and 20) during sleep and a more marked day-night contrast after EYT. These changes in the direction of greater complexity and altogether greater HRV during the night after EYT could reflect more refreshing sleep and explain the reduction in fatigue symptoms. This effect has been shown by the shift of the degree of variability and complexity represented mainly by the nonlinear indices (Table 1). A supporting result was found by Stein et al. [18], who observed that exercise training increases total heart rate variability in normal older adults and that the most marked alterations are in nocturnal heart rate. This development is supported by the change of the ULF index. The reduction of the ultralow oscillations (ULF) during the night reflects more relaxed sleep with a smaller number of movements, while the changes during the day displayed a slightly modified HRV. These changes during sleep may be one explanation for the already published lesser fatigue symptoms [4, 13, 19] after the YET training. Furthermore, the complexity measures of symbolic dynamics (pW232 and pTH7), short-term symbolic dynamics (mean_huegel), and MSE (scale 2 and scale 20) [16] show a comparable pattern. The index mean_huegel is increased during the night after the 6-week course of YET training which means that the probability of occurrence of this word type is much more pronounced (representing increased short-term regulation related to increased vagal activity). In contrast, the index pTH7 is decreased during the night after EYT. This means that word types with a probability of occurrence of more than 7% are reduced. This causes an increase of other word types, again leading to increased complexity. The MSE indices scale 2 and scale 20 (Figures 2 and 3) confirm this development of increased complexity [15]. Even if the indices of all scales from MSE are generally increased during the night after EYT (Figure 2(b)) representing a general increase in complexity through all time scales, the differences between day and night, however, only showed a significant increase after EYT in scale 2 (Figure 3(a)). This could be a marker for increased short-term vagal activity (see also HFn). Interestingly, An et al. [20] could show that the state of relaxation after cyclic meditation results in parasympathetic dominance in women, as evidenced by the increased HF component of the RR-interval series. The fact that the dynamics of heart rate variability is different in women as compared to men has also been seen. The observed significant increase in complexity (sampling entropy) after the practice when the subjects report a sense of calmness and well-being.

Thus, in the context of the published data of the study, the subjects appear to sleep better at night and be more alert during the day resulting in a better quality of life [11, 21]. This finding is supported by the study of Grant et al. [22] who observed that exercise interventions lead to a significant increase in vagal influence during supine, rising, and standing.

It seems likely that negative influences such as daytime stress which can reduce vagal activity during the night [23] and shift the sympathovagal balance towards sympathetic predominance were reduced by the EYT intervention in this study. This was demonstrated by better stress coping abilities after the EYT intervention [4, 13, 19]. This is an interesting finding as we know the alarmingly negative association of stress with cardiovascular health [24]. Stimulation of vagal activity during the night has also been shown for other interventions, such as cyclic meditation [25]. 

The strength of this analysis is that the changes shown to result from the EYT training are confirmed by various methods of HRV analysis. The already published improvements in health-related QoL, stress coping, and fatigue symptoms [4, 13, 19] are substantiated by these analyses. The effects within the group examined are robust and present an overall plausible picture. 

The precise mechanism of action of EYT is still difficult to explain. Conceptionally, EYT attempts to utilise basic physiological rhythmic processes and to stimulate these through speech, speech rhythms transformed into movement, and special movement sequences. Although we are still a long way far from understanding its mode of action, this training method nevertheless appears to have significantly positive clinical effects. The results obtained to date should be further examined in clinical studies and particularly also in patients suffering from cancer, fatigue, or other forms of chronic stress.

An effect of this kind has not previously been described for other mind-body interventions but may be of general interest. “Sleep is the best medicine” is an old saying which contains a lot of truth. Many illnesses can be caused or negatively influenced by lack of sleep or an unhealthy sleep-waking rhythm [26–30]. In addition to promoting the development of cancer or having a negative impact on its course, disturbed sleep has even been shown to compromise processes of immunological regulation [31–34]. There is a close functional relationship between sleep and the autonomic nervous system [35]. The cyclic pattern of healthy sleep is subject to active regulation by the autonomic nervous system and shows a highly regulated rhythmic picture of chronobiological processes in direct interaction with autonomic functions of the cardiovascular system such as blood pressure, heart beat, and respiration [34, 35]. Status changes in the transition from one sleep phase to another have a marked impact on parameters of HRV or blood pressure variability, for example [36, 37]. Sleep disturbances are often accompanied by changes in the circadian rhythms [38, 39]. This has been studied for various neurological diseases [40, 41] and for hypertension [42]. In cancer patients, disturbances of the circadian rhythm are associated not only with fatigue symptoms [38, 39], which compromise patients' quality of life, but also with a significantly worse prognosis [43]. Therefore, it is of general clinical interest to search for therapies [44] or interventions leading to better sleep or a better chronobiologic [38, 39] pattern of the activity of the autonomous nervous system. 

### 4.1. Limitations of the Study

For organizational reasons, it was not possible to randomize the study. Therefore, we cannot rule out the possibility that the EYT group might have contained preferentially subjects who believed that they could benefit from a treatment aimed at stress reduction and improvement of quality of life. However, this only affects the comparison of the psychometric data, not the HRV analysis. The fact that the study lacked an appropriate control intervention such as light aerobic training remains a weakness of the study. 

Furthermore, we are aware of the influence of respiratory rates on HRV parameters.

Unfortunately, it was not feasible to monitor respiration for 24 hours in this setting. It was beyond the scope of this study to have completely standardized conditions as we were interested in real-life effects. Because of limited availability of male subjects, we have an unbalanced male/female ratio in this study with a majority of women. Therefore, the subsequent analysis was based only on the data of women. However, this fact is supported by findings of other groups. The difference in heart rate variability between men and women has been commented on before [22, 45], where it was observed that baroreflex responsiveness is attenuated and vagal activity is augmented in women compared with men. Because the LF component of the heart rate variability reflects, in part, the baroreflex-mediated control of the heart rate, women tend to display a lower value of power in the LF spectrum of the heart rate variations leading to a more predominant vagal modulation. An et al. observed this behavior demonstrating a parasympathetic dominance in women (in contrast to men). Furthermore, Uusitalo et al. [46] could not find significant changes in cardiac and vascular autonomic regulations with regular exercise training in 140 men. These findings support the hypothesis that men and women show a different reaction on exercise and stress and therefore should be investigated separately.

We performed the multivariate ANOVA analysis to figure out time and phase dependencies. Here, we found only significant phase dependencies and no significant time dependencies and interactions between time and phase; even a clear trend in several indices could be observed in time dependencies and interactions. This is not surprising because the results from multidimensional two-factor ANOVA have to be considered with caution due to the relative small number of cases. Further, about half of the indices had to be transformed to normal distribution leading to additional side effects. 

Finally, in an ongoing study, we will increase the number of included subjects enabling us to prove definitely the dependency of the changed day and night time differences on the EYT outcome performing a two-factor ANOVA with repeated measures. 

## 5. Conclusion

The results of this study show that a 6-week course of EYT training can have a positive impact on the circadian rhythm of the heart rate. There is a shift in HRV during the night towards a more vagal-dominated rhythm with a greater complexity corresponding better to the physiological pattern at night. The day-time rhythms of HRV also changed in terms of a trend towards a more typical day-time pattern. Therefore, in summary, we see an overall improvement in the circadian rhythm which could provide an explanation for the improvement of QoL, fatigue symptoms, and stress coping. This treatment should be further investigated in patients with chronic illnesses. Patients with cancer and fatigue syndrome, in particular, could benefit from the treatment.

## Figures and Tables

**Table 1:** Descriptive statistics of day/night time before and day/night time after EYT (mean value ± standard deviation) of HRV indices of day/night time before and day/night time after EYT including significance (*P*) of differences (*P < 0.05, **P < 0.01, and ***P < 0.001; n.s.: not significant).

Method	Index	Day time before EYT	Night time before EYT	*P*	Day time after EYT	Night time after EYT	*P*
HRV	meanNN	719.9645 ± 97.5070	946.1659 ± 110.8942	∗∗∗	722.7145 ± 97.5392	924.8230 ± 112.5868	∗∗∗
	sdNN	85.4704 ± 22.0300	81.5635 ± 21.8195	n.s. (0,54)	86.6286 ± 27.7480	74.4476 ± 22.5824	n.s. (0,07)
	ULF	1126.2409 ± 883.6143	845.5170 ± 897.9354	n.s. (0,33)	1147.2612 ± 919.5328	586.1914 ± 557.9539	∗∗
	LF/HF	6.6243 ± 3.6584	5.7644 ± 4.6834	n.s. (0,38)	6.3557 ± 3.1467	5.1150 ± 4.1323	n.s. (0,06)
	LFn	0.8458 ± 0.0612	0.7860 ± 0.1207	n.s. (0,06)	0.8434 ± 0.0590	0.7598 ± 0.1479	∗
	HFn	0.1254 ± 0.0612	0.2140 ± 0.1207	n.s. (0,06)	0.1566 ± 0.0590	0.2402 ± 0.1479	∗
	(ULF + VLF)/P	0.8695 ± 0.0665	0.8186 ± 0.0994	n.s. (0,07)	0.8648 ± 0.0799	0.7948 ± 0.0915	∗∗∗

SD	pW232	0.0090 ± 0.0038	0.0113 ± 0.0050	n.s. (0,08)	0.0077 ± 0.0045	0.0132 ± 0.0077	∗∗
	pTH7	2.8261 ± 0.8341	3.5217 ± 1.0388	∗∗	2.9130 ± 0.9493	3.1304 ± 0.9197	n.s. (0,26)

STSD	peak	0.0580 ± 0.0168	0.0661 ± 0.0229	n.s. (0,06)	0.0524 ± 0.0157	0.0693 ± 0.0313	∗∗

MSE	scale 2	1.1177 ± 0.3088	1.2196 ± 0.3020	n.s. (0,30)	1.1318 ± 0.2786	1.3841 ± 0.2733	∗∗
	scale 20	1.5635 ± 0.2595	1.2889 ± 0.4138	∗∗	1.5730 ± 0.3626	1.4349 ± 0.2847	n.s. (0,22)

*P*: significance of differences: *P < 0.05, **P < 0.01, and ***P < 0.001; n.s.: not significant.
